# Retraction Note: Confidence level based complex polytopic fuzzy Einstein aggregation operators and their application to decision-making process

**DOI:** 10.1038/s41598-025-86717-1

**Published:** 2025-01-21

**Authors:** Khaista Rahman, Mohammad Khishe

**Affiliations:** 1 Department of Mathematics, Shaheed Benazir Bhutto University Sheringal, Dir Upper, 1800 Pakistan; 2 Department of Electrical Engineering, Imam Khomeini Naval Science University of Nowshahr, Nowshahr, Iran; 3 Innovation Center for Artificial Intelligence Applications, Yuan Ze University, Taoyuan, Taiwan; 4 Applied Science Research Center, Applied Science Private University, Amman, Jordan

Retraction of: *Scientific Reports* 10.1038/s41598-024-65679-w, published online 02 July 2024

The Editors have retracted this Article after concerns about a significant overlap with a different paper that was under submission at the same time but has different authorship. The Authors were unable to provide a suitable explanation of the discrepancy in authorship. The authorship of this article, therefore, cannot be verified.

Mohammad Kishe disagrees to this retraction. Khaista Rahman has not responded to correspondence from the Editor about this retraction.

